# Strategies to prevent, curb and eliminate biofilm formation based on the characteristics of various periods in one biofilm life cycle

**DOI:** 10.3389/fcimb.2022.1003033

**Published:** 2022-09-21

**Authors:** Ruixiang Ma, Xianli Hu, Xianzuo Zhang, Wenzhi Wang, Jiaxuan Sun, Zheng Su, Chen Zhu

**Affiliations:** Department of Orthopedics, The First Affiliated Hospital of University of Science and Technology of China (USTC), Division of Life Sciences and Medicine, University of Science and Technology of China, Hefei, China

**Keywords:** bacterial infection, biofilm formation, plankton adhesion, quorum sensing, nanomaterial, combined therapy, antibiotics resistant

## Abstract

Biofilms are colonies of bacteria embedded inside a complicated self-generating intercellular. The formation and scatter of a biofilm is an extremely complex and progressive process in constant cycles. Once formed, it can protect the inside bacteria to exist and reproduce under hostile conditions by establishing tolerance and resistance to antibiotics as well as immunological responses. In this article, we reviewed a series of innovative studies focused on inhibiting the development of biofilm and summarized a range of corresponding therapeutic methods for biological evolving stages of biofilm. Traditionally, there are four stages in the biofilm formation, while we systematize the therapeutic strategies into three main periods precisely:(i) period of preventing biofilm formation: interfering the colony effect, mass transport, chemical bonds and signaling pathway of plankton in the initial adhesion stage; (ii) period of curbing biofilm formation:targeting several pivotal molecules, for instance, polysaccharides, proteins, and extracellular DNA (eDNA) *via* polysaccharide hydrolases, proteases, and DNases respectively in the second stage before developing into irreversible biofilm; (iii) period of eliminating biofilm formation: applying novel multifunctional composite drugs or nanoparticle materials cooperated with ultrasonic (US), photodynamic, photothermal and even immune therapy, such as adaptive immune activated by stimulated dendritic cells (DCs), neutrophils and even immunological memory aroused by plasmocytes. The multitargeted or combinational therapies aim to prevent it from developing to the stage of maturation and dispersion and eliminate biofilms and planktonic bacteria simultaneously.

## Introduction

Bacteria have much longer history than human beings, paleontologists believe that bacteria arose from the collision and reaction of various inorganic and organic materials in the ancient extreme earth environment billions of years ago, and therefore have some stronger environmental adaptability than we human. These advantages in bacterial adaptability are reflected not only in their constantly gene mutations, bacterial spores, capsule and flagella, but also in getting together to counteract disturbances, known as biofilms: a complex multicellular and animated lifestyle, which is one of the most wide-spread and predominant living pattern on earth. The terminology biofilm was firstly documented in the fields of microbiology on environmental technology, and was not introduced into medicine for the first time until 1982 by Dr. Costerton, as he observed that microbial community aggregated on the surface of a cardiac pace-maker catheter and formed biofilm matrix (Marrie et al., 1982). The self-produced extracellular polymeric substances (EPS) are mainly comprised of polysaccharides, proteins, eDNA and overexpressed reductive glutathione (GSH) (Flemming et al., 2016). The all above are responsible for protecting the bacterium from deleterious environmental conditions, including antimicrobial agents and the physiological immune system (Teirlinck et al., 2018). Additionally, the constituents of EPS are greatly diversified, relying on nutritional availability, host conditions and physical stresses strength. A couple of studies have recently validated that eDNA may be an important and essential component of the biofilm matrix by adding DNase to the surface of mature biofilms *in vitro* experiments and detecting structure damaged.

Besides, the biofilm-induced inflammation can lead to DNA damage in host cells, thus increasing the risk of cancer (Parsonnet, 1995; Jobin, 2014; van Elsland & Neefjes, 2018). Microbial biofilm-related persistent infections and tissue damage are becoming extremely difficult to treat and eradicate permanently by conventional antibiotic therapy due to their intrinsic resistance to antibiotic agents (sensitivity only up to 1/1000 of previous) compared to plankton in the flowing milieu (Sundin et al., 2020).

The mechanisms of bacterial antibiotic resistance, such as efflux pumps, antibiotic-modifying enzymes and gene mutations, are well investigated, but they are limited to planktonic bacteria (Sun et al., 2020; Xiu et al., 2022). Novel evidence indicates that bacteria situated inside biofilms have lower oxygen and nutrient levels, which means a lower micro-metabolic level (Zhang et al., 2019). Exactly this resulted in the enhancement of tolerability to antibiotics that chiefly target metabolically active bacteria. Furthermore, biofilm can spread as a source of infection to nearby healthy sites. The switch between single planktonic and complex biofilms is a key factor for bacteria to trigger different infectious diseases, including prosthetic joint infections (PJIs), infective endocarditis and osteomyelitis (Peng et al., 2019). Bacteria are always vulnerable to antimicrobial agents once they scatter out of biofilms, which indicates that the antibiotic resistance of bacteria in biofilms isn’t acquired through mutations. The explicit mechanism has not been utterly researched and has been the topic of comprehensive investigation until now. Especially in a surgical implant, once the biofilm formed, there is very few viable treatments or managing options available (Xie et al., 2018). Although bacterial antigen can spur generation of antibodies, they are unlikely to slay bacterium inside biofilms efficiently. On contrast, they accompany with unfavorable immune complexes to nearby healthy cells expect for monoclonal antibodies (Raafat et al., 2019). The persistence of biofilms can lead to medical implants fault or biological material deterioration as well. Above all, these are exactly why bacterial biofilm related diseases, such as osteoarthritis, valvulitis, rhinitis, cystitis, vaginitis, periodontitis, specific pneumonia, otitis, etc., of which implant-associated infections are the most common, are currently the culprits of chronic infections bringing tremendous survival hazards to mankind (Luis Del Pozo, 2018). While bacterial biofilms develop over the exterior of surgical implants (for example, a prosthesis of the knee joint), they tend to aggregate inflammatory compositions and secrete metabolic substances, resulting in an acidic multi-radical microenvironment, which contributes to the failure of wound healing (Rumyantceva et al., 2021). Most secondary surgeries for implant removal in clinical practices are linked closely to biofilm development by *Staphylococcus aureus (S. aureus)*. Especially, *methicillin-resistant Staphylococcus aureus (MRSA)* and *Vancomycin-resistant Staphylococcus aureus (VRSA)* have caused grave damages to many people (Sun et al., 2020). We argue that the treatment of biofilms should be focused on their adhesion and formation phases, since biofilms in this phase have not yet developed their distinctive resistance properties. As well, since new biofilms usually originate from the dispersion of previous existing biofilms, blocking dispersion of the previous biofilm also falls under the key aspect of preventing the formation of new biofilms. Thus, methods to interfere with mature biofilm dispersion will also be discussed in our review as a complementary part.

Methods conventionally used for biofilm treatment have their limitations:

(i) Surgical sterile precautions and techniques aim to prevent infection, but often fail to achieve sterilization in the treatment of acute contaminated wounds and the curative rate of debridement is not entirely satisfying (Gilbert-Girard et al., 2020); (ii) Eluting devices and wound by using anti-microbial or antibiotic solutions to inhibit early planktonic adhesion to infixed devices, while effective, can sometimes compromise the function of certain devices and accelerates the emergence of antibiotic-resistant bacteria; (iii) Injecting agents to disrupt established biofilms and eliminate microbes within them is not a common clinical treatment, but often causes cause secondary damage to the patient without guarantee efficacy.

As of today, removing the contaminated equipments continues to be the most effective therapeutic option, which is extremely painful and brings not only multiple surgeries, but also considerable harm and financial burden to patients. We therefore review numerous new therapeutic strategies that differ from the above conventional approaches, since these strategies are characterized by precise and even targeted therapies based on the characteristics of each period of biofilm formation. Here, we systematize the therapeutic strategies into three main periods precisely: (i) period of preventing biofilm formation: interfering the colony effect, mass transport, chemical bonds and signaling pathway of plankton in the initial adhesion stage; (ii) period of curbing biofilm formation: targeting several pivotal molecules, for instance, polysaccharides, proteins, and eDNA *via* polysaccharide hydrolases, proteases, and DNases respectively in the second stage before developing into irreversible biofilm; (iii) period of blocking biofilm dispersion to prevent the sprouting of new biofilms: applying novel multifunctional composite drugs or nanoparticle materials cooperated with ultrasonic, photodynamic, photothermal and even immune therapies. With all these curative strategies, the structure and function of biofilms are compromised by different mechanisms and efficiencies across different phases of biofilm formation. Herein, we review the characteristics of each period of biofilm formation and the corresponding innovative healing therapies in detail.

## How do bacteria develop a biofilm?

**Figure 1:**
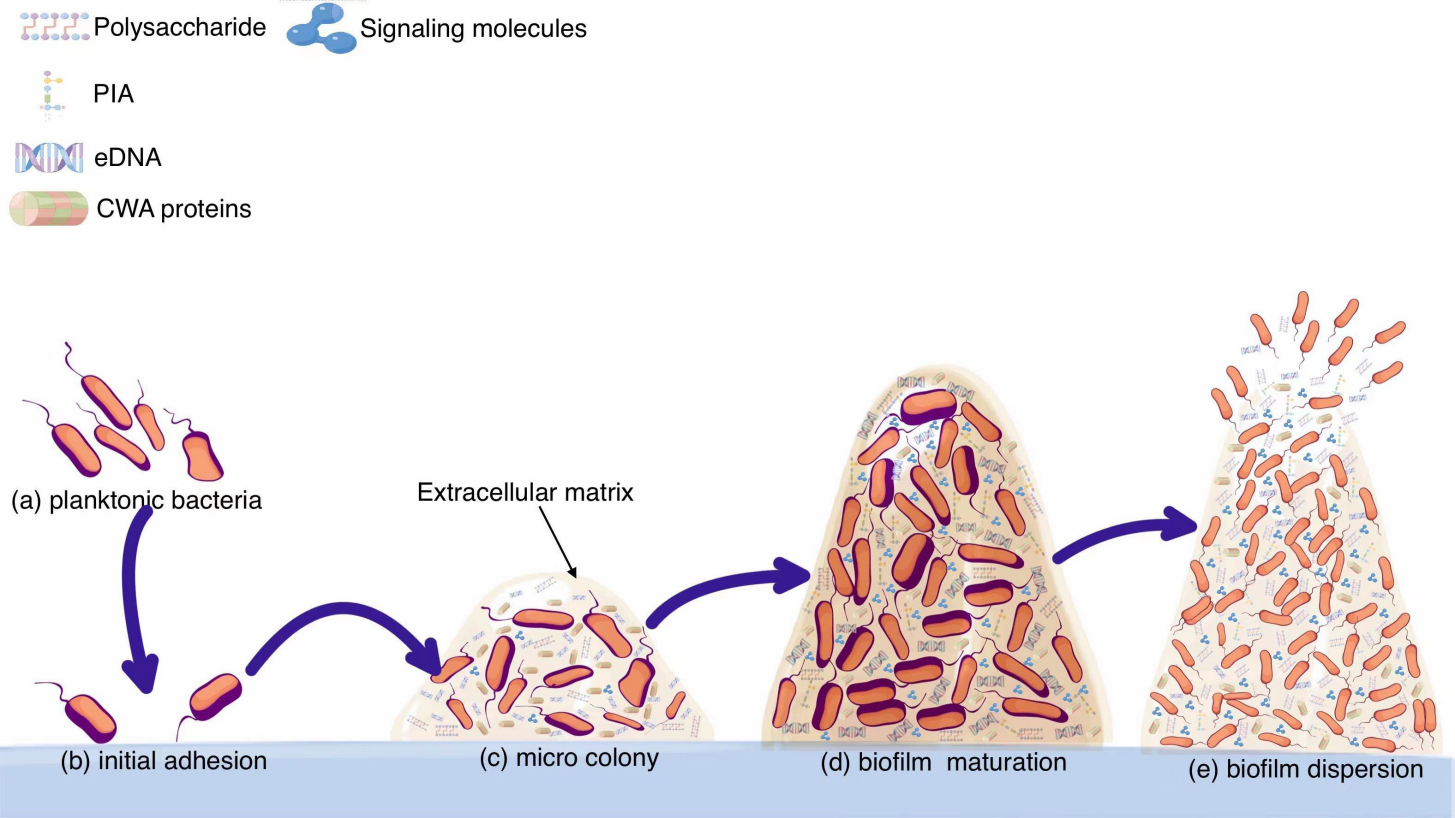
From planktonic bacteria to mature biofilms. **(A)** Planktonic bacteria invade the wound and swim randomly. **(B)** Planktonic bacteria adhere to the implant surface, which is a reversible process. From forming microcolonies **(C)** to developing into mature biofilms **(D)**. **(E)** Biofilm breaks down and bacteria spread outside.

As a dynamic biological system, biofilm has evolved a large number of networks to coordinate biofilm formation in different outer circumstances. Formation steps of biofilms are closely modulated through multiple modulatory chains (Tolker-Nielsen, 2015). The physiological status of bacteria, subtle variations in ambient settings and the ever-changing events inside the bacterial colonies are all underlying signaling factors that impact on biofilm formation. Many works have been done towards identifying and characterizing bacterial physiological activity at the cellular level, and these technological advances will contribute to understanding the complex biofilm-forming mechanisms and developing novel specific therapeutic approaches (Qu et al., 2020). Activating biofilm generation by regulatory pathways will guarantee that bacteria do not establish biofilm under adverse circumstances. It is evident that the establishment of a steady biofilm is regulated by multiple factors, and part of these engage as two-component signaling pathways (Landry et al., 2018). The overall processes of biofilm formation are shown in **Figure 1**.

### The initial adhesion stage

Reversible adhesion is usually initiated by the non-specific forces (van-der Waals, Brownian motion and electrostatic forces) haphazardly and then followed by the irreversible adhesion on the surface *via* a variety of different factors and mechanisms to form a complicated three-dimensional colony that is known as a biofilm (Dixit et al., 2019). Bacteria can sense contacts with surfaces and accordingly adjust gene expression to facilitate stable cell-surface interactions. Superficial adhesion of planktonic cells primarily contact substrates of the host organism during infection with various adhesins, such as fibrinogen, fibronectin and collagens such as fibronectin binding proteins (FnBPs) and fibrinogen-binding clumping factors (Clfs) (Palmqvist et al., 2005).

Environmental signals inside the biofilm allow bacteria to efficiently colonize their preferred environment. Previous studies have demonstrated that under extreme conditions of nutrient enrichment or deprivation, bacteria are more likely to be in the planktonic mode, as this provides them with greater access to nutrients and new habitats. Satisfactory meld of implants with host tissues or plankton is the cornerstone of implant success. At the moment the biomaterials implanted, the competition for surface between host cells and bacteria starts. Once planktonic bacteria preempt to adhere on the surface, the innate immune system alone can hardly prevent the biofilm formation (Dapunt et al., 2020). Thus, the architectural features of the implant itself have a critical impact on the occurrence of bacterial infections.

The bacterial flagella and pilus are the two main power sources in the planktonic state, which are motivated by ion inflow *via* membrane-spanning motor complexes. The flagella are generally several folds longer than the bacteria itself and is the most powerful motor to drive the plankton towards the surface of the implants. When the bacteria sense that the surrounding environment is favorable for their proliferation, the flagella will anchor to the surface, otherwise they will drive the bacteria away to find better habitats (Guttenplan & Kearns, 2013). *S. aureus* have no flagella and therefore may rely on bacterial pilus and other signaling agents to mediate attachment to a surface.

The shear stress, which constantly varies with positions and fluid flows, acts as another key factor affecting the initial adherence of the plankton. Increased shear stress enhances the polysaccharide intercellular adhesin (PIA) expression of the bacteria to accommodate the shear stress (Schaeffer et al., 2016). Lectins also facilitate and consolidate the surface adhesion process of planktonic bacteria, especially the effect of surfactants to reduce the surface tension of the contact surface is critical to stabilize the later formed micro-colonial structure (Shi et al., 2021). Besides, cell wall anchoring (CWA) proteins promote planktonic bacteria to bind with surface matrixes (Bannoehr et al., 2011; Milles et al., 2018). The bacterial FnBPs, Clfs, CWA proteins and serine-aspartate repeat (Sdr) proteins are collectively referred as microbial surface components recognizing adhesive matrix molecules (MSCRAMMs), which can modulate interactions between host extracellular matrix components and microbes (Flick et al., 2013). Such recognizing adhesion molecules are strain-specific, for example the adhesin involved in diffuse adherence (AIDA-I) is involved in the initial diffuse adhesion of E. coli, while P. aeruginosa is rely on the lectins (Charbonneau et al., 2007). The adhesion regulatory factors are summarized in **Table 1**.

**Table 1:** The main planktonic adhesion regulatory factors.

Planktonic adhesion-related factors	Effects	Ref.
**Physico-chemical factors** Flagella and pilus	Related to the initial adhesion and terminal dispersion of planktonic bacteria.	(Gonzalez et al., 2018)
Wall teichoic acids (WTAs)	Wall teichoic acids acids (WTAs) located on the bacterial surface are crucial in maintaining bacterial membrane integrity, escaping host defenses, and mediating toxicity.	(Yin et al., 2019)
hydrophobic and electrostatic interaction van-der Waals, Brownian motion Shear stress	The main driving force for the movement of planktonic bacteria from irregular mobility patterns to initial adhesion. Promote bacterial adhesion to the surface of clinical biomaterials. Supports initial attachment of bacteria to the contact surface and increases adherence; Brownian motion provides impetus for planktonic bacteria. The lower the density of bacteria the greater the effect of Brownian motion. The effect on planktonic bacteria depends on the infectious position of the and the implant surface topography. Bacteria exposed to shear stress distributes mechanical stress along the pili.	(Carniello et al., 2018) (Chen et al., 2014) (Zhang et al., 2015) (Geisel et al., 2022) (Krsmanovic et al., 2021)
**microbial surface components recognizing adhesive matrix molecules(MSCRAMMs)** FnBPA and FnBPB CWA proteins	(i) drive attachment to the surface of surgical an implant and boost intercellular communications, leading to biofilm formation; (ii) The activity of FnBPs depends on the integrity of SarA. A vital virulence factor for survival in symbiotic states and invasive infections.	(Dziewanowska et al., 2000) (O'Neill et al., 2008) (Foster et al., 2014)
ClfA and ClfB	(i) ClfA binds to FnBPA to enhance bacterial virulence and facilitate biofilm formation.	(Claes et al., 2018)
SdrC, SdrD, SdrG, and SdrE	(ii) The binding of FnBPB and ClfB with similar affinity promoted the firm binding of *S. aureus* to tissue cells. (i) Binds fibrinogen, collagen and kerati; (ii) All are pathogenic antigens of *S. aureus*. (iii) SdrC enhances bacterial attachment to plastic surfaces, but whether it is associated with the hydrophobicity of the proteins is still ambiguous. (iv) In blood, SdrD increases virulence and vitality of *S. aureus*.	(Towell et al., 2021) (Gogoi-Tiwari et al., 2015) (Feuillie et al., 2017)
autolytic enzymes AtlA, homologous protein AtlE	(i) Help intial attachment to abiotic surfaces like polystyrene or glass (ii) Increased expression promotes bacterial autolysis, leading to higher emission of eDNA.	(Kaplan et al., 2012) (Rosman et al., 2021) (Mashruwala et al., 2017)
Sortase	(i) Sortase is prevalent in most Gram-positive bacteria and has a vital effect in the interaction of *S. aureus* with fluid environment. (ii) Mutations in the Sortase-dependent pathway can anchor surface CWA proteins to the outer membrane of Gram-positive bacteria. (iii) Mediate the motility of pili.	(Jiang et al., 2013) (Sugimoto et al., 2017) (Bhat et al., 2021)
PIA lectins	Participates in the formation of biofilms, bacterial virulence and drug resistance Keep bacteria increasing in biofilm by binding to exopolysaccharide Psl in EPS.	(Wu et al., 2017) (Passos da Silva et al., 2019)

### Developing into irreversible biofilm by secreting EPSs

In the process of biofilm formation, bacteria regulate the interconnection of adhesion factors within the matrix *via* a distinctive info-communication webs. Ultimately, a sophisticated three-dimensional tower-like biofilm structure is formed. As soon as the bacterial settle on the surface successfully, the bacteria begin to multiply and excrete all kinds of extracellular substances to envelop themselves, which marks the beginning of irreversible adhesion and opens the portal to biofilm formation. While numerous studies have been conducted on the composition of bacterial extracellular secretions, the exact components remain uncertain. The major known compounds consist of (i) extracellular polysaccharide, the main component of which is polysaccharide intercellular adhesin (PIA) produced by icaADBC locus with strong ability to promote adhesion; (ii) eDNA, which is presumed to be generated by bacteria either by active apoptosis or passive autophagy to facilitate the survival of bacterial community (Valle et al., 2019). And eDNA can stabilize the biofilm matrix and enhance gene exchange between bacteria, and may even be a key factor in promoting bacterial gene mutations (Lei et al., 2019). It also triggers immune responses when eDNA interacts with host immune cells, but the ultimate effect of such immunity is anti-inflammatory, which is not conducive to biofilm clearance, instead leading to refractory and chronic infections; (iii) diverse proteins that mediate adhesion and signaling communications, e.g., Bap, the surface protein of *S. aureus*, can build amyloid scaffolds to stabilize bacterial accumulation (Taglialegna et al., 2016). And some bacteria secrete amyloid curli, a protein that can bind to eDNA to forge firm fiber-like polymers inside biofilms and react with host cells to yield immunogenic complexes that can activate a variety of immune cells, including DCs characterized by the production of cytokines such as type I interferon (IFN-I) (Yan et al., 2020); (iv) phospholipids, which can be covalently coupled to peptidoglycan to form wall teichoic acids (WTAs) to increase bacterial attachment towards implant by binding with fibronectin or to cytoplasmic membranes to form lipophospholipids. Besides, it has also been reported that bacterial secreted virulence agents and ribosomal proteins also contribute to the stabilization of biofilms. Bacteria possess a specific surface sensing system, and the system can be activated within minutes after the bacteria adhere to the surface.

### Quorum-sensing system

Regulation of QS is almost throughout biofilm formation. The high density of bacteria within biofilms enables a variety of signaling communications among them, which relies on bacterial density to coordinate signaling transductions (Wu et al., 2022). And QS can link diverse elements that are responsible for enabling the necessary virulence and metabolism to maintain the survival of bacteria (Carnes et al., 2010).

The accessory gene regulator (agr) is a bioregulator of Gram-positive pathogens, especially for *Staphylococcus* (Kavanaugh & Horswill, 2016; Jenul & Horswill, 2019; Qu et al., 2020). Agr QS can directly regulate the virulence and adhesion of *S. aureus* (Hardy et al., 2019). With low expression of agr, *S. aureus* tends to form biofilms by secreting more intercellular adhesins and reducing the secretion of toxins (Valliammai et al., 2019). Conversely, increased intracellular expression of AgrD is followed by the secretion of autoinducer peptide (AIP) (Kim et al., 2017).The detailed composition of Agr varies between strains. The extracellular AIP concentration increases proportionally with increasing cell density. Once the local AIP concentration meets a threshold level, AIP binds to AgrC to activate the AgrC-AgrA two-component system. The activated Agr system restrains the expression of AtlE (an essential adhesive protein involved in biofilm construction), thereby inhibiting biofilm formation and enhancing the expression of virulence factors such as pore-forming toxins and tissue-degrading enzymes, reinforcing the bacterial resistance to external harmful substances such as ROS.

Further tests *in vitro* proved that the addition of AIP can activate the agr-mediated breakage of *S. aureus* biofilm, and the bacteria will restore to the planktonic state, thus completing the biofilm life cycle. This may be due to the fact that Agr activation can lead to elevated levels of staphylococcal proteases that cleave bio-membrane proteins and disrupt intercellular interactions within the biofilm, and that proteases can also be applied to the biofilm. There are also matrix-degrading materials, such as dispensin B, which can cause biofilm disintegration by weakening the structural integrity of the biofilm matrix. Activation of the agr system induces the expression of phenol-soluble modulins (PSM), a low molecular weight pore-forming toxin with surfactant-like properties. S-ribosylhomocysteine lyase (LuxS) is involved in production of autoinducer 2 (AI-2).

There are certain alternative regulatory pathways that are also relevant to the formation of biofilms, which allow bacteria to micro-modulate their response to ever-changing environmental conditions and moderate biofilm formation. Intracellular second messengers, such as cyclic dinucleotides (cDN), c-di-GMP, have multiple physiological and immunomodulatory abilities in bacteria, often enhancing bacterial virulence and biofilm formation. Besides, cyclic dimeric (3′→5′) GMP (c-di-GMP) induces the production of interferon-gamma (IFNγ) to prolong the host type I interferon immune response.

A dual regulatory CpxA/CpxR signaling system relies on the outer membrane protein NlpE, acting as a direct sensor of E.coli for surface contact. The Cpx pathway becomes activated in response to the interaction of E.coli with hydrophobic surfaces, accelerating the oscillation of pilus to drive bacteria moving. EnvZ/OmpR signaling system is another dual component signaling pathway used in E. coli, can be activated under elevated osmolarity to strengthen the adhesion of bacteria to biological surfaces to counteract the adversity. If under excessive osmotic pressure, EnvZ/OmpR signaling will be overtaken by a negatively regulated system, which means the bacterial cells will remain in the planktonic mode and freely migrate to more favorable conditions.

**Figure 2:**
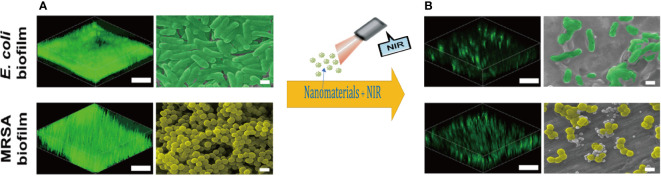
Three-dimensional reconstructions of the fluorescence-labeled (scale bars, 30 µm.) and Scanning electron microscope images (scale bars,1 µm) of **(A)** mature *E. coli* and *MRSA* biofilms; **(B)** damaged biofilm treated with photodynamic nanomaterials. Copyright ^©^ 2022, Advanced science.

Mature biofilms may seem to be thick and homogeneous cell cushion structures, but they are complex architectures feature with a hydraulic tunnel structure in order to keep nutrients inflowing and wastes out just as **Figure 2** presented. Such a complex structure is not exclusively controlled by physical factors, such as shear. In fact, several modulatory variations have already influenced the integral deepness and structure of the mature biofilm. And c-di-GMP is another ubiquitous second messenger with potent immunomodulatory properties in biofilm, stimulating innate immunity and regulating biofilm formation, planktonic motility, and virulence. Besides, c-di-GMP can bind to a wide range of receptors, including enzymes, splice proteins, transcription factors and nucleoprotein switches, enhancing the recruitment of neutrophils, macrophages, natural killer cells and even DCs to kill bacteria. Low doses nitric oxide (NO) signaling has been shown to stimulate specific phosphodiesterases (PDEs), triggering c-di-GMP degradation and concomitant diffusion of bacterial biofilms. *In vitro*, low doses of NO are the primary dispersion-driven mediator, and release of NO contact with biofilms can be targeted to enhance antimicrobial efficacy while limiting potentially toxic effects on target tissues. In E. coli, high levels of c-di-GMP enhance the adhesion of planktonic bacteria and promote biofilm formation, while it drives biofilm rupture at low levels, allowing the internal bacteria to spread out. In *S. aureus*, this second messenger role is assumed by c-di-AMP. A number of small regulatory RNAs (sRNAs) regulate targets associated with bacterial colony behavior, including QS (Malgaonkar & Nair, 2019).

### Advanced biofilm formation and its secondary dispersion

**Figure 3:**
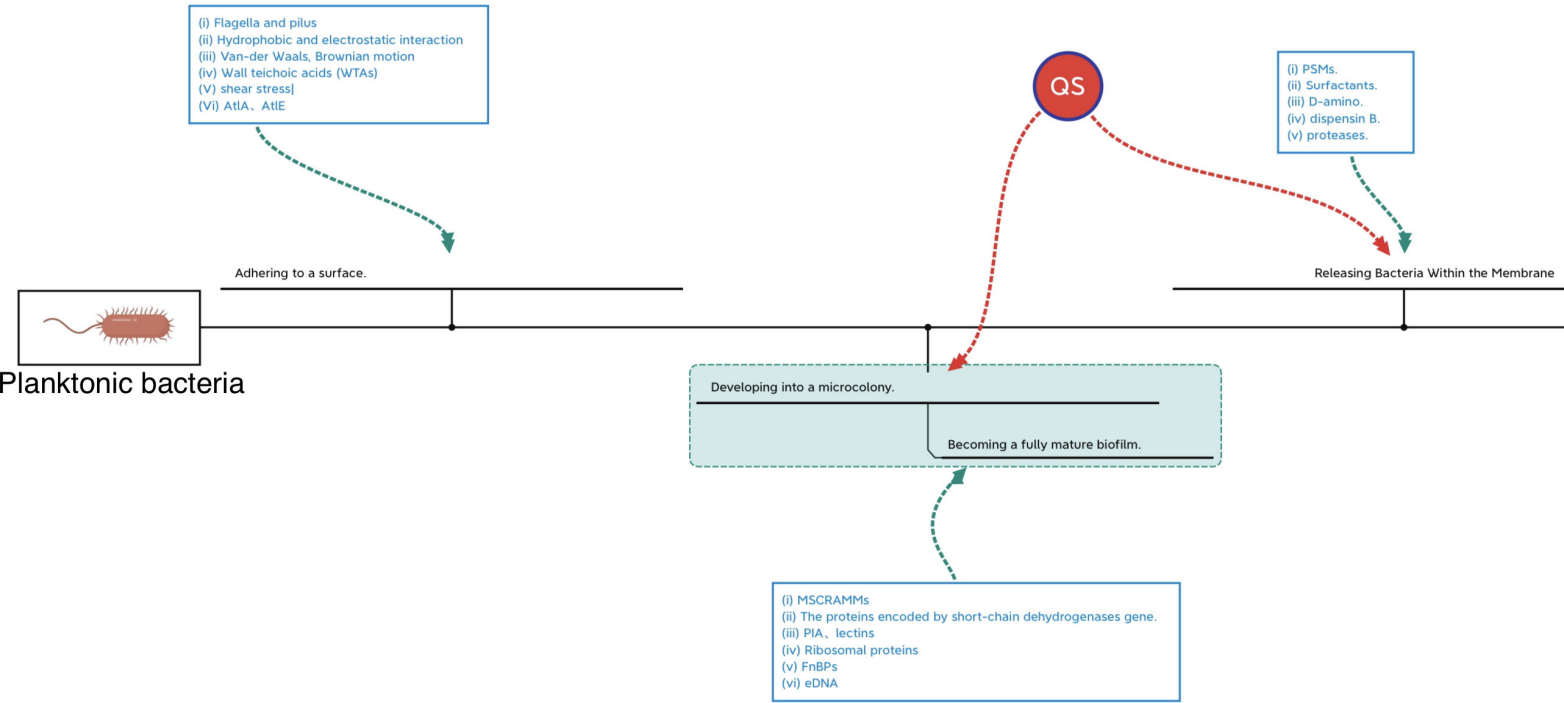
Regulating networks in different periods of biofilm formation.

The formation of microcolonies by planktonic bacteria is a critical step in biofilm formation. A microcolony is usually a three-to-five-layer-deep colony of bacterial cells that evolves as bacterial cells adhere to the surface. The development of stable interactions between single bacteria and the surface alone is insufficient to form the microcolony; Some destructive factors such as nucleases and PSMs break down the biofilms leading to catastrophic secondary mass release of bacteria and the spread of inflammation. Bacterial spreading during biofilm rupture is mainly driven by proteases and PSMs to degrade and disrupt the biofilm matrix synergistically (Hommes et al., 2021). Each stage of biofilm formation is influenced by different factors just as shown in **Figure 3**. Factors associated with biofilm maturation and diffusion are summarized in **Table 2**.

**Table 2:** Factors associated with biofilm maturation and diffusion.

Factors	Effects	Ref.
ribosomal proteins SspB、SspA FnBPs Bap eDNA PSMs Surfactants D-amino Psl	Facilitating biofilm stabilization and maturation. SspB is a crucial factor of bacterial virulence. Allowing cells to interconnect during biofilm accumulation. One of the essential proteins for biofilm formation. Promoting biofilm formation through a mechanism isolated from the biofilm-associated polysaccharide PNAG. (i) Mediating the interaction of biofilms with other matrix components for resilient stress; (ii) Maintaining biofilm stability; (iii) Inducing host immune defense. Stimulating diffusion behavior of mature biofilms. Inhibiting the continued growth of biofilms and shifting biofilm towards breakage. Induce the release of amyloid fibrils and prevent biofilm formation; A common signal for biofilm breakdown. Protects bacteria from the direct effects of antibiotics.	(Guilhen et al., 2016) (Bonar et al., 2016) (Foster, 2016) (Matilla-Cuenca et al., 2020) (Mottola et al., 2016) (Peterson et al., 2013; Lei et al., 2019; Sultan et al., 2019) (Periasamy et al., 2012) (Zhao et al., 2013; Dane et al., 2014; Ueda et al., 2019) (Kolodkin-Gal et al., 2010; Leiman et al., 2013) (Welp & Bomberger, 2020)

## Preventing biofilm formation

Traditional treatments for bacterial infections fail to clear biofilm related chronic infections *in vivo*, and the abuse of high dosages of antibiotics for a prolonged period is obviously inadvisable, since in many cases it induces drug resistance and further biofilm development (Yan & Bassler, 2019). Most current therapies target the acute exacerbation of infection caused by the release of planktonic bacteria but do not focus on the characteristics of biofilm growth to prevent its formation before it fully matures. Until now, surgical excision of the infected implant and wound debridement are still the principal method of biofilm elimination, but this is not always feasible because of the large physical trauma and the high risk of complications (Beebout et al., 2021). Apart from the mechanisms such as efflux pumps and genetic mutations which exist in usual drug-resistant bacteria, bacteria within biofilms have some specific tolerant mechanisms. Antibiotics may function in inhibiting the further progression of biofilms. Yet, they are unable to kill them. It has long been assumed that an essential mechanism of biofilm resistance is due to the complex matrix components such as eDNA and polysaccharides contained in biofilms to sequester drugs. However, further studies have shown that the reality is not so easy. Tetracyclines can infiltrate biofilm to cover all bacteria within the E. coli biofilm in less than 10 minutes at the cost of losing their killing capacity. Thus the biofilm does not simply rely on blocking the antibiotic to exert resistance. It may be a more important mechanism to slow the penetration of the antibiotics and giving the bacteria enough time to develop resistance (Grygorcewicz et al., 2020). In addition small colony variants (SCVs) inside biofilms are strongly associated with persistent and recurrent osteomyelitis and implant-associated infections, especially in *S. aureus* biofilms (Tuchscherr et al., 2015). β-lactamases that degrade antimicrobials are also present in the biofilm matrix, further preventing the drug from reaching the cells inside. Therefore, we present innovative approaches based on targeting on each period of clinical biofilm formation, such as the application of QS inhibitors (QSI) targeting the QS system, drugs that interfere with bacterial metabolic pathways, reagents that can destroy the biofilm components and even combine with activated immune cells such as DCs and macrophages to kill bacteria.

### Prevent initial adhesion

Planktonic bacteria reproduction and migration is essential for subsequent bacterial colonization in dynamic fluidic milieu. Despite planktonic bacteria possessing robust mobility, the concentration of agents required to kill them tends to be only one thousandth of the biofilm (Fan et al., 2017). Therefore, targeting planktonic bacteria is critical for early containment of biofilm formation.

Interfering the signaling transductions between bacteria. Disrupting QS-related signaling pathways to restrain the biofilm associated gene expression is considered as an advisable approach to curb biofilm formation. Previous studies have well recognized that genetic variations and bacterial transcriptome can be modulated by suppressing QS (Cole et al., 2018). QS governs the expression of bacterial virulence factors, and therefore blocking QS can largely diminish the virulence of bacteria (Piewngam et al., 2020). QSI act primarily through five interactions with QS signaling molecules: (i) inhibition of synthesis; (ii) acceleration of degradation; (iii) competition for receptor sites; (iv) inhibition of gene expression; (v) removal of AIs (Kalia et al., 2019). For example, mutations in the LasR/pqsH/cdpR gene appear to influence QS in biofilm, which have inspired the creation of gene-targeted drugs (Kuang et al., 2020). While urea in urine has been reported to suppress bacteria by interfering with the QS signaling communication (Kang et al., 2017). Due to this, some experts argue that QS is almost negligible in urinary tract infections (Feltner et al., 2016; Kang et al., 2017; Cole et al., 2018). The occurrence ascribed to downregulation or silencing of the QS system is called quorum quenching (Rajput & Kumar, 2017). Most QS rely on acyl-homoserine lactone (AHL), AIP and AI-2 for communication (Kim et al., 2017; Kim et al., 2018). Therefore, QS can be modulated by using analogs of the above signaling molecules (Billerbeck et al., 2018).

As already mentioned previously, host reactions with FnBPs and ClfA are decisive drivers of bacterial virulence, and therapies based on intervening FnBPs and Clfs may be another effective way to prevent initial adhesion (Flick et al., 2013).

Autophagy originally refers to the degradation behavior of long-lived proteins and organelles in eukaryotic cells upon binding to lysosomes. Recently, autophagy has been found to be linked to infectious conditions and plays a vital role (Huang et al., 2011). During bacterial infection, autophagy can be activated by a variety of host factors and pathways, including the formation of autophagosomes around the targeted bacteria and then transferring these pathogens to lysosomes for further degradation (Sparrer et al., 2017). However, bacteria have evolved multiple strategies to interfere with autophagic signals to avoid autophagy, and in some cases bacteria can even utilise autophagy to benefit their survival (Holla et al., 2014). Autophagosomes begin at the phagocytic assembly site (PAS), an endoplasmic reticulum (ER) sub-structural domain rich in phosphatidylinositol 3-phosphate (PtdIns3P) (Huang & Brumell, 2014). A set of autophagy-associated (ATG) proteins is a critical player in precisely regulating autophagy (Moreau et al., 2014). Studying the interactions among bacterial factors and ATG proteins will be emphasized in the future for the treatment of bacterial infections. In physiological conditions, complement protein C3 is deposited on invasive pathogens, and Matthew T Sorbara et al. utilized C3 complement to trigger antimicrobial cell autophagy intracellularly (Sorbara et al., 2018). The role of selective macroautophagy in targeting intracellular pathogens for lysosomal degradation is a relatively well-established direction as well as the immune effect of xenophagy (Kimmey & Stallings, 2016). Although autophagy mainly eliminates planktonic and intracellular bacteria, this is prospective for the prevention of biofilm formation (Wu & Li, 2019).

Modulation of the surface where bacteria adhere with the increasing popularity of surgical implants, bacterial adhesion infection is becoming more common. Therefore, it is an innovative approach to make anti-bacterial implant by modifying their surface characteristics. The application of biocides coated on the surface of implants is an effective inhibitor of biofilm formation, but has not been used on a large scale in clinical practice due to toxicity and other limitations. Natural and effective biofilm inhibitor coatings to alter the surface of implants have become the focus of new research. Multi-species biofilms formed by *Escherichia coli* and *S. aureus* can be effectively eliminated by coating with various silver coatings (Hou et al., 2020; Lee et al., 2021). Nanotopologies kill bacteria within minutes by penetrating their membranes with mechanical force. Duy H K Nguyen et al. developed a 35 nm diameter silica nanopillar to rupture *P. aeruginosa* and the sterilizing rate was up to 85%, but less than 10% for *S. aureus*, indicating the effectiveness and non-broad-spectrum limitations of nanomaterial topography for antibacterial activity (Nguyen et al., 2019). Nanostructured mechanical sterilization is assumed to be realized by piercing the phospholipid bilayers of microbes. Titanium surfaces with pocket-type nanostructures killed nearly 50% of the initially adherent bacteria and effectively prevented potential recurrence (Cao et al., 2018).

### Disrupt biofilm

Antimicrobial peptides (AMPs), which target bacterial membranes, are generally amphiphilic cationic micromolecules consisting of 10-50 amino acids with broad-spectrum antimicrobial capacity and retaining high sensitivity to metabolically dormant bacteria in biofilms (Jiang et al., 2022). AMPs are commonly applied in combination with antibiotics and other anti-biofilm compositions to combat biofilms. As an integral member of innate immunity, the host defense peptide (HDP) directly targets planktonic cells and exhibits both anti-biofilm and host-directed immunomodulatory activity. Recently, there have been increasing reports referring to the emerging anti-biofilm properties of HDPs that are different from those previously recognized. Meanwhile, the synergistic effects with other therapeutic approaches such as antibiotics are making HDPs shine in the fight versus biofilms. Daniel T. Cohen et al. synthesized antimicrobial peptide-vancomycin complexes by using coupling chemistry and proved the broad-spectrum antimicrobial effect of the complexes exceeding vancomycin *in vitro* (Cohen et al., 2019).

The exploitation of antimicrobial nanomaterials as a substitute for antibiotics is currently a hot spot in medicine with promising prospects. The micro diameter of the nanomaterials is pivotal to their functions. However, a considerable number of nanoparticles tend to aggregate in solution, which may limit their application with photothermal therapy (PTT)/Photodynamic therapy (PDT). An effective approach to overcome this barrier is to use polymers (e.g., PEG, and BSA) to carry them as well as to conduct the necessary surface functionalization to enhance dispersibility and biocompatibility (Jiang et al., 2022).

In the last century PTT has entered a phase of rapid development and is widely used to fight against cancer and microbial infections (Cheng et al., 2015; Qi et al., 2018; Deng et al., 2020; Yang et al., 2020). PDT requires applying specific wavelengths of light upon photosensitizers to produce reactive oxygen species (ROS) in the specific inflammatory microenvironment, which can lead to bacterial damage and even death. However, excessive amounts of ROS may also harm normal tissues and be detrimental to wound recovery, which is one of the challenges to be overcome for novel photothermal therapies (Mitsunaga et al., 2011). As regards how PDT kills bacteria hidden within the biofilm, it may be due to the destruction of the channels engaged in the transport of nutrients to the core region and the loss of biofilm integrity (Xie et al., 2018). In recent years, many nanomaterials possess both excellent antibacterial properties and good biocompatibility, some of which have strong killing effect on drug-resistant bacteria. However, these photothermal effects cause certain damage to normal tissues while exerting antibacterial properties, which is not conducive to wound recovery. Therefore, the preparation of low-cost, rapid and effective antibacterial and tissue repair nano-composite materials has become one of the research frontiers. From a synergistic perspective, the combination of PDT and PTT maximizes efficacy and meanwhile minimizes side effects (Zou et al., 2019).

Triggered gas-releasing nanoparticles that serve as gas donors or gas carriers to apply as a substitute to conventional antibacterial drugs in medicine. Bactericidal gase s, such as hydrogen sulfide (H2S), NO and hydrogen (H2), are emerging to provide for efficient gas therapy for infectious illnesses combined with an excellent biosafety *in vivo* (Su et al., 2022). Gas therapy is frequently synergized with near infrared (NIR) stimulated phototherapies (e.g., PTT or PDT). NO gas exhibit powerful anti-biofilm efficacy primarily through mediating bacterial DNA damage. Besides, NO has the potential to eradicate bacterial biofilms in host by stimulating M1 polarization of macrophages. In contrast, the anti-inflammatory properties of carbon monoxide (CO) and NO can mitigate the inflammation-related response in the anti-biofilm process. H2S is another DNA-damaging mutagen, but it exerts an anti-inflammatory effect by facilitating the polarization of M2 macrophages, i.e., it has the advantage of both NO and CO, which can diminish the negative effects of PTT while improving the therapeutic effect and eliminating the biofilm. Up-to-date researches focus more on the smart responsive gas release function of nanoparticles to achieve spatio-temporal regulation, thus improving the sustainability and controllability of treatment. Nano-carrier MoS2-BNN6 can not only effectively treat *Escherichia* coli and *S. aureus*, but also provides precise control of NO release by irradiating with 808 nm laser. Then, Mos2-BNN6 destroys cell membranes through PTT/NO, which synergistically induced ROS. At the same time, MoS2 also accelerated the oxidation of GSH under 808nm irradiation, destroyed the balance of antioxidant in bacteria, shortened the treatment time, and achieved efficient bacterial inactivation within 10 minutes (>97.2%). In addition, mos2-BNN6 nanocarriers can release NO at low concentrations after infection control, promoting tissue repair (Gao et al., 2018). AI-MPDA, an integrated phototherapy nanoplatform composed of L-Arginine (L-ARG), indocyanine green (ICG) and mesoporous polydopamine (MPDA), provides PTT and PDT *via* generating ROS to induce the l-ARG cascade catalytic release of NO (Yuan et al., 2020). In April 2021, a Chinese scholar reported a dual-acting nanoparticles, deoxyribonuctinase I (DNase I)-CO-mesoporous polydopamine nanoparticles (MPDA NPs), featured controlled release of CO gas by NIR. DNase-CO@MPDA NPs that can effectively eliminate methicillin-resistant *MRSA* biofilms were made by encapsulating the photosensitive CO donor FeCO in MPDA NPs and then covalently anchoring DNase I on the surface of MPDA NPs. under NIR irradiation, the released DNase I can degrade eDNA in biofilm and disrupt the outer sphere of biofilm. Simultaneously, CO gas is released, which can fully infiltrate the damaged biofilm and thoroughly eradicate the residual bacteria. Finally, NIR-activated DNase I-CO@MPDA NPs promotes healing of infected skin wounds by locally accelerating CO release, which may be related to CO increasing mitochondrial biogenesis and driving mitochondrial increased ATP production (Yuan et al., 2020). Jun Li et al. published an exogenous antibacterial agent composed of Zinc-doped Prussian blue (ZnPB), which can accelerate the emission and infiltration of ions into microbes by local heat induced by photothermal effect, resulting in changes in cellular metabolic changes. Besides, ZnPB upregulates the expression of genes involving in cell proliferation, promoting collagen deposition and facilitates wound healing (Li et al., 2019).

Gallium (Ga)-based nanoparticles has been tested to eradicate biofilms in mice and achieved an excellent result *in vivo* and *in vitro*. The PDT effect induced by ICG-Ga NPs destroys bacterial membranes and accelerates the endocytosis of Ga3+, which substitutes ions in bacteria with Ga3+ blocks bacterial iron metabolism, exerting a synergistic effect of bacterial killing and biofilm destruction. The ultra-small size of ICG-Ga NPs can be removed rapidly by the kidney, guaranteeing the biocompatibility (Xie et al., 2021). Another recently proposed article of the exact opposite mechanism of anti-inflammatory action of classical Ga ions (Ga3+) *via* delivering Ga nanodroplets (GNDs) to lipopolysaccharide-induced macrophages. GNDs exerted a selective inhibition of NO generation without interfering with the accumulation of pro-inflammatory agents by disrupting the synthesis of inducible NO synthase in activated macrophages through up-regulation of eIF2α phosphorylation levels, without disturbing Fe homeostasis (Zhang et al., 2022). A lipophilic Ga complex, Ga2L3(bpy)2, has both Ga (disruption of iron metabolism) and ligand effects (production of ROS) in the fight against drug-resistant bacteria (Wang et al., 2021).

As a star material of the century, graphene and its derivatives have got a splash in the medical field due to its favorable biocompatibility and antibacterial potential (Cao et al., 2021). Compared to other nanocomposites, graphene is cheap, environmentally friendly and easy to manufacture. The graphene oxide (GO) can produce mechanical damage and oxidative stress to biofilms (Palmieri et al., 2017). An innovative protective coating based on graphene and hydrogels has been proposed as new anti-biofilm coating material to prevent microbial adhesion (Cacaci et al., 2020). GO films can be used as biocompatible sites for bacterial adhesion on their surfaces (Ming et al., 2020). Graphene-based nanomaterials (GBNMs), with their unique structures and extraordinary physicochemical properties, have been intensively investigated and widely used in many biomedical fields to improve bactericidal efficacy and reduce adverse effects in the treatment of bacterial biofilm (Wang et al., 2022).

### Combined immunotherapy

It is known that biofilms preserve their dominance by suppressing host immunity, and thus modulating body immunity to regain a proactive status is a pressing medical issue (Heim et al., 2020). The studies of the interaction between bacteria and the host immune system to adapt and develop strategies for the treatment of bacterial infections is an extremely promising new path. Intrinsic immunity has evolved with the body over tens of thousands of years and has a well-established and elaborated system, and the skillful activation of some beneficial immune capacity can be pivotal and essential in combating biofilms (Campos & Zampieri, 2019). The most prospective is the activation of *in situ* immunity *via* advanced biomimetic nanomaterials that act as vaccines to produce a powerful and sustained antimicrobial effect with self-immune cells, such as neutrophils, macrophages, and DCs (Kimkes and Heinemann, 2020).

As antigen-presenting cells, DCs are core in stimulating and mediating the host immune system, and numerous previous works have revealed that DCs perform a key function in initiating antigen-specific immunity and immune tolerance when facing with bacterial infections (Sabado et al., 2010; Cao et al., 2013). The cGas/sting-IFN1 pathway, which is known to sense accumulation of nucleic acids and induce inflammatory responses, is crucial in the immune response of DC cells. The cGas/sting-IFN1 pathway has made significant progress in the treatment of cancer (Thomas et al., 2017; Alicea-Torres et al., 2021). With the presence of biofilm, various inflammatory mediators produced by the bacterial inflammatory response can upregulate DC-Sting by enhancing DCs to engulf bacteria *via* targeting C-type lectin (CTL), which is specific in the recognition and capture of pathogens by DCs and the subsequent generation of effector T cell (Ramakrishna et al., 2019). However, it has been reported that *in vitro* co-culture assays of biofilms, DCs maturation got stunted and CTL expression decreases significantly, which means the powerful STING pathway may be inactivated (Srikanth et al., 2019). Eventually, DCs convert from antigen-capturing cells to antigen-presenting cells, leading to adaptive immune dysregulation and persistent growth and random invasion of microorganisms in DCs (El-Awady et al., 2015). Kaya, E et al. reported that significant activation of CD56(+) CD3(-) natural killer cells was observed after co-culture of peripheral blood mononuclear cells (PBMC) and bacterial biofilms. Natural killer (NK) cells exert not only direct antibacterial effects, but also interact with other immune cells through cytokines such as perforin and interferons to generate indirect antibacterial activity. Thus, transmigration of NK cells to local infectious sites may be a promising option (Schmidt et al., 2016)

As effector cells of the innate immune system, neutrophils are involved in a variety of immune inflammatory response processes. Neutrophils exert extracellular neutrophil traps (NETs) through the cell death program of suicidal NETosis. The reticular DNA structure released by the extrusion of genomic DNA is a siege for invading pathogens and preventing dissemination (Dhanesha et al., 2020). Nuclear and granule protein (histone G and proteinase 3, etc.) will soon eliminate the trapped bacteria by binding to the reticular DNA (Dhanesha et al., 2020). *S. aureus* biofilms skewed neutrophil to neutrophil NETs formation *via* the combined activity of the leukocyte inhibitor Panton-Valentine leukocyte inhibitor and γ-hemolysin AB causing the antibacterial activity of NETs to be ineffective in eliminating biofilm and even exacerbating biofilm infection (Bhattacharya et al., 2018). Current research aims to treat refractory bacterial infections by targeting neutrophil development and proliferation to regulate the accumulation of neutrophils at the site of infection and to mitigate the deleterious effects of NETs (Nemeth et al., 2020). Augmentation of neutrophil numbers and function by adding G- CSF, inhibiting CXCR4 and blocking CD47-SIRPα interactions may be a therapeutic approach to enhance the function of neutrophils in infections (Németh et al., 2020).

Macrophages function centrally in antimicrobial immunity, with recognition, phagocytosis and bactericidal capabilities (Serezani et al., 2009). There is growing evidence that *S. aureus* biofilm infection in PJIs establishes an immunosuppressive environment associated with myeloid-derived suppressor cells and M2-macrophages (Peng et al., 2017). Therefore, in the battle against biofilms, it is crucial to convert macrophages from the immunosuppressive M2 type to the antimicrobial M1 type (Lei et al., 2019). Gold nanoclusters (Au NCs) coupled with mercaptopyrimidines can be used as highly effective nanoantibiotics that can target and kill bacteria. The antibacterial mechanism is disruption of biofilm structure, such as eDNA induction of ROS production and macrophage polarization (Zheng et al., 2018). He et al. forged an antimicrobial polymer polyhexamethylene biguanide (PHMB) hybridized with gold nanoparticles (Au NPs) platform (PHMB@Au NPs). PHMB@Au NPs exhibit superior synergistic effect to enhance both photothermal bactericidal effect under NIR irradiation and tissue repair by converting macrophages from M1 type to M2 type (He et al., 2022). The mechanisms and period of action of the various treatments are summarized in **Table 3**.

**Table 3:** Summary of therapeutic approaches to inhibit biofilm formation.

Therapeutic method	Mechanism of effects	Stage of biofilm formation				Ref.
**QSI and other signal blockers**		Initial adehesion	Colony formation	Maturation	Dispersiona and second adhesion	
RsaL	Reduces QS signals and securing homeostasis by performing a counter role to LasR.	✕	□□	✔□	✕	(Kang et al., 2017) (Cole et al., 2018) (Balaban et al., 2007) (Wang et al., 2017; Sethupathy et al., 2020)
Urea	Interfering with the quorum sensing pathway, inactivating it.	✕	□□	✔□	✕	
RIP	Downregulates TRAP/AGR system and disorders biofilm formation.	✕	✔□□	✔□	✕	
Indole	Perturbs bacterial QS and inhibiting biofilm formation and virulence factor emission.	✕	✔□□	✔□	✕	
AHL lactonase	Degrades or inactivates AHL.	✕	✔□	✔□	□	(Liu et al., 2018)
**Implant topography** Nanotopological structural surface	Prevent bacterial adhesion, mechanical stress sterilization.	✔□	✕	✕	✔□	(Yi et al., 2019; Khalid et al., 2020; Velic et al., 2021; Zheng et al., 2021)
**Nanomaterials** Gallium (Ga)-based (i) ICG-Ga NPs (ii) Ga nanodroplets	Gallium(III) exhibits superior multi-targeted antibacterial activity. Maintaining pro-inflammatory antibacterial microenvironment and reduce NO release.	✔□□	✔□□	✔□□	✔□	(Vinuesa & McConnell, 2021; Li et al., 2022)
Graphene-based (i) graphene oxide (GO) (ii) Magnetic Graphene-Based Sheets Mn-based (i) hybrid membrane@MnOx@PpIXP (ii) Manganese salts	improving bactericidal efficacy and reduce adverse effects in the treatment. Capture and destroy bacteria by high frequency magnetic field. Activating cellular and humoral adaptive immunity against bacterial infections. As an immune adjuvant to activate cytotoxic T cells.	✔□□	✔□□	✔□□	✔□□	(Dasari Shareena et al., 2018) (Hardiansyah et al., 2020) (Lin et al., 2022) (Zhang et al., 2021)
		✔□	✔□□	✔□□	✔□□	
**Antimicrobial agents** host defense peptide (HDP) Human Calprotectin (CP) D-tyrosine DNase Artificial monoclonal antibodies	Multifunctional effectors of the innate immune system with antibacterial and pleiotropic immunomodulatory properties. Chelates iron, manganese, zinc and other trace elements necessary for bacterial metabolic survival, leading to disruption of bacterial metabolism. Inhibits bacterial biofilm formation and triggers decomposition with species specificity. Degrades eDNA in biofilms. Specifically targeting certain components of the biofilm surface.	✔□□	✔□□	✔□	✔□	(Yu et al., 2020) (Nakashige et al., 2015) (Yu et al., 2016) (Novotny et al., 2016)
		✔□	✔□□	✔□	✔□□	
		✔□	✔□□	✔□	✔□	
		✕	✔□	✕	✔□	
		□□	✔□	✕	□□	
**Immunotherapy** Dendritic cells (DCs) (i) nanovaccines synergized with adoptive DC transfer. (ii)Activation of cGas-STING-IFN I Neutrophils (i) neutrophil-derived drug delivery systems. Macrophages (i) MW-responsive engineered pseudo-macrophages. (ii) CuFe5O8 nanocubes (NCs).	(i) Inducing IgA production. (ii) Initiating specific immunity such as T-cells by directly phagocytosing bacteria and presenting antigens. killing bacteria directly after activation. M1 polarization promotes the elimination of biofilms. M2 polarization was not conducive to biofilm removal.	✔□	✔□□	✔□□	□□	(Akazawa et al., 2018; Mi et al., 2021) (Xiao et al., 2021) (Zhou et al., 2022) (Kim et al., 2021; Wang et al., 2021) (Rao et al., 2020; Fu et al., 2021) (Guo et al., 2020)
		✔□	✔□□	✔□	□□	
		✔□	✔□□	✔□□	□□	

✔✔ □□ means good effect; ✔ □means a little effect; ✕ means little effect.

## Conclusion

There are numerous and disparate approaches to prevent, treat, and eradicate biofilms in clinical practice, but ultimately there is a lack of comprehensive and uniform understanding of the overall physio-pathological process of biofilm-induced inflammation, resulting in a dearth of effective treatments and alternative strategies for serious bacterial infections and chronic refractory biofilm inflammation that occur after surgical operations. The necessity to reasonably tailor the treatment by understanding the characteristics of the biofilm itself. The therapeutic approaches we present above are based on three major components. Firstly, we aim to preempt to thwart the ability of planktonic bacteria to form further biofilms during the migration and initial surface adhesion phase. Secondly, we design to destroy the developing seeds through a combination of therapeutic approaches between initial adhesion and biofilm maturation stages. Thirdly, we plan to stop the spread of planktonic bacteria at the time of biofilm dispersal to contain the development of biofilms.

Conventional antibiotic therapy is often insufficient to eradicate biofilm infections. Rather than the singular therapy of the direct treatment of biofilm formation or proliferation, we recommend combination therapies that are intelligently targeted according to the characteristics of their material composition at each stage of formation. The advantages of these special therapies have been described in detail above, but there are still some shortcomings that need to be overcome.

Numerous studies on interfering QS systems and other signaling pathways have been reported, but there is still a lack of sufficient animal models to confirm the applicability of such methods *in vivo*. Investigations of QSI *in vivo* need to be further enhanced and optimized to reach the level of clinical application to better overcome severe inflammations arising from antibiotic-resistant pathogens (Seghal Kiran et al., 2016). Disruption of biofilm structure is also a viable and effective strategy, but it often requires combination of sensitive antibiotics to eradicate the remaining bacteria within the biofilm. Antimicrobial topological surface have been explored for years, but it functions mainly in the early adhesion stage and the underlying mechanisms are still unclear and the diverse effects of topographies such as micropillars, rows and concaves, especially at the nanoscale, need to be explored further (Echeverria et al., 2020).

While local drug injections and ultrasound therapy are still commonly used to combat implant infections, these alone are not sufficient to deal with recalcitrant drug-resistant bacteria, and surgical debridement is often ineluctable eventually. Hence, combining treatments depending on the period of biofilm formation described in this review will be necessary to eradicate the biofilm utterly.

The application of nanomaterials has opened a new chapter in the field of antibacteria, but the impacts of the materials themselves on the host remains a thorny issue that cannot be avoided. Admittedly, although nanotechnology and membrane-coatings enable materials to be more biocompatible and powerful, the practicality and durability of such composite materials hinder their further application. The emerging biomimetic nano vaccine technology further refines the nano antimicrobial therapy. As artificial nanocomposites wrapped by host cell membranes, biomimetic vaccines possess excellent biocompatibility and different immunophysiological characteristics based on the coating membranes. For example, nanomaterials covered with macrophage membranes can target bacterial infections autonomously through toll-like receptors (TLRs) on the coating (Lin et al., 2022). Moreover, membranes from erythrocytes, platelets, tumor cells and other cells are available for generating bionic antibacterial materials and deserve further investigation (Xiang et al., 2021).

Despite current comprehensive and significant advances in biofilm therapy, the popularity of “biofilm” has kept the term at the forefront of any literature on bacterial infections. Therefore, in the future, we need to further develop the above-mentioned biofilm treatment strategies and combine them with each other to perform a comprehensive therapeutic system from prevention to eradication and eventually to prevent recurrence, so as to completely stop any possible development of biofilm and to eliminate inflammatory infections in their cradle.

## Author contributions

RM, XH wrote the manuscript. CZ revised the review. All authors contributed to the article and approved the submitted version.

## Funding

This work was supported by the National Natural Science Foundation of China (Grant No. 81871788), the Key Research and Development Program of Anhui Province (No. 202004j07020013 and 2022e07020017), the Natural Science Foundation of Anhui Province (Grant No. 2108085QH319), the Fundamental Research Funds for the Central Universities (Grant No. WK9110000173), the National Natural Science Foundation of China (82102586); the Fundamental Research Funds for the Central Universities (WK9110000155).

## Conflict of interest

The authors declare that the research was conducted in the absence of any commercial or financial relationships that could be construed as a potential conflict of interest.

## Publisher’s note

All claims expressed in this article are solely those of the authors and do not necessarily represent those of their affiliated organizations, or those of the publisher, the editors and the reviewers. Any product that may be evaluated in this article, or claim that may be made by its manufacturer, is not guaranteed or endorsed by the publisher.

## Glossary

**Table:**

Agr	Accessory gene regulator
AHL	Acyl-homoserine lactone
AMPs	Antimicrobial peptides
AIDA-I	Adhesin involved in diffuse adherence
AI 2	Autoinducer 2
AIP	Autoinducer peptide
ATG protein	Autophagy-associated protein
BAP	Biofilm Associated Protein
BSA	Bovine serum albumin
CO	Carbon monoxide
CWA	Cell wall anchoring
Clfs	Clustering factors
CTL	C-type lectin
c-di-GMP	Cyclic dimeric (3′→5′) GMP
cDNs	Cyclic dinucleotides
DCs	Dendritic cells
ER	Endoplasmic reticulum
eDNA	Extracellular DNA
EPS	Extracellular polymeric substances
FnBPs	Fibronectin binding proteins
Ga3+	Ga ions
GNDs	Ga nanodroplets
Ga	Gallium
GSH	Glutathione
GO	Graphene oxide
GBNMs	Graphene-based nanomaterials
HDP	Host defense peptide
H2	Hydrogen
H2S	Hydrogen sulfide
ICG	Indocyanine green
ica	Intercellular adhesion
IFN-γ	Interferon-gamma
L-ARG	L-Arginine
MPDA	Mesoporous polydopamine
MRSA	Methicillin-resistant Staphylococcus aureus
MSCRAMMs	Microbial surface components recognizing adhesive matrix molecules
NK cells	Natural killer cells
NIR	Near infrared
NETs	Neutrophil extracellular traps
NO	Nitric oxide
PBMC	Peripheral blood mononuclear cells
PAS	Phagocytic assembly site
PSM	Phenol-soluble modulins
PtdIns3P	Phosphatidylinositol 3-phosphate
PDEs	Phosphodiesterases
PDT	Photodynamic therapy
PTT	Photothermal therapy
PEG	Polyethylene glycol
PHMB	Polymer polyhexamethylene biguanide
PIA	Polysaccharide intercellular adhesion
PJIs	Prosthetic joint infections
QSI	QS inhibitors
QS	Quorum-Sensing System
ROS	Reactive oxygen species
Sdr	Serine-aspartate repeat
SCVs	Small colony variants
sRNAs	Small regulatory RNAs
TLRs	Toll-like receptors
LuxS	S-ribosylhomocysteine lyase S. aureus Staphylococcus aureus
IFN-I	Type I interferon
US	Ultrasonic
VRSA	Vancomycin-resistant Staphylococcus aureus
WTAs	Wall teichoic acids
ZnPB	Zinc-doped Prussian blue
